# Characteristics associated with COVID-19 vaccine hesitancy

**DOI:** 10.1038/s41598-022-16572-x

**Published:** 2022-07-20

**Authors:** Liyousew G. Borga, Andrew E. Clark, Conchita D’Ambrosio, Anthony Lepinteur

**Affiliations:** 1 Department of Behavioural and Cognitive Sciences, University of Luxembourg, 4366 Esch-sur-Alzette, Luxembourg; 2 Paris School of Economics – CNRS, 75014 Paris, France

**Keywords:** Vaccines, Human behaviour

## Abstract

Understanding what lies behind actual COVID-19 vaccine hesitancy is fundamental to help policy makers increase vaccination rates and reach herd immunity. We use June 2021 data from the COME-HERE survey to explore the predictors of actual vaccine hesitancy in France, Germany, Italy, Luxembourg, Spain and Sweden. We estimate a linear-probability model with a rich set of covariates and address issues of common-method variance. 13% of our sample say they do not plan to be vaccinated. Post-Secondary education, home-ownership, having an underlying health condition, and one standard-deviation higher age or income are all associated with lower vaccine hesitancy of 2–4.5% points. Conservative-leaning political attitudes and a one standard-deviation lower degree of confidence in the government increase this probability by 3 and 6% points respectively. Vaccine hesitancy in Spain and Sweden is significantly lower than in the other countries.

## Introduction

To arrest the spread of the COVID-19 pandemic, a significant share of the population needs to be immune to the virus. It is commonly considered that the safest way of achieving this goal is by vaccination^1^. Within 1 year of the onset of the pandemic, the European Commission authorised the use of a number of vaccines against COVID-19 in the EU, following evaluations carried out by the European Medicines Agency. As of December 3rd 2021, 82% of adults aged 18 or over in the EU had received at least one vaccine dose, and 77% were fully vaccinated. Up-to-date statistics are available via the European Centre for Disease Prevention and Control’s vaccine tracker (available at: https://www.ecdc.europa.eu/en).

Even though cases of COVID-19 are currently on the rise again worldwide (predominantly among the unvaccinated^2,3^) and new virus variants are appearing, many individuals remain hesitant to get the jab. There are concerns that this COVID-19 vaccine hesitancy will pose substantial risks for both those who delay or refuse vaccination and the wider community, as it renders the threshold necessary for herd immunity harder to reach^4^.

Vaccine hesitancy is defined by the World Health Organisation as a delay in the acceptance or refusal of vaccination despite the availability and accessibility of vaccination services^5^. A large body of epidemiological and public-health literature has considered the complex and context-specific causes of vaccine hesitancy in general, and hesitancy regarding COVID-19 vaccines in particular^6,7^.

Most existing work on vaccine hesitancy uses data from surveys that were conducted prior to the approval and widespread distribution of COVID-19 vaccines, and asked whether individuals would agree to receive vaccines once they became available: as such, individuals report their hypothetical choices. In the UK, hypothetical COVID-19 vaccine hesitancy is higher for women, the younger, and those with lower levels of education^8^. In Australian data, women, those living in disadvantaged areas, those who thought COVID-19 risks were overstated, those holding more populist views, and the more religious were more likely to be hesitant or resistant^9^. In nationally-representative data from Ireland and the UK, those resistant to a COVID-19 vaccine were less likely to obtain information about the pandemic from traditional and authoritative sources^10^. Similar research has been conducted in the US^11,12^, Portugal^13^, Japan^14^, France^15^, and Israel^16^. The common predictor of vaccine hesitancy and delay across this research seems to be a general feeling of distrust towards either the government or health-service responses to the pandemic, or the efficacy or necessity of the COVID-19 vaccine itself.

We here provide new evidence to add to the scant literature about revealed or actual vaccine hesitancy (*i.e.* the refusal to take the currently-available COVID-19 vaccines), which has mostly focused on the US^17–19^. To our knowledge, we are the first to document the prevalence and predictors of actual vaccine hesitancy in six European countries. Our analysis covers France, Germany, Italy, Spain, Sweden and Luxembourg, using unique harmonised real‐time data from the COME-HERE (COVID‐19, MEntal HEalth, REsilience, and Self‐regulation) longitudinal survey collected by the University of Luxembourg in June 2021. We run multivariate regressions to establish the most-important individual and societal predictors of revealed vaccine hesitance. We then compare our results to those in the previous hypothetical-choice literature to establish whether both the levels and predictors of stated and revealed vaccine hesitancy differ. By doing so, our results help to identify the groups that are the most hesitant in real life: these are the groups that policy-makers may wish to target in order to increase vaccination rates and the prospect of reaching herd immunity.

## Methods

### Data: COME-HERE

The COME-HERE longitudinal survey is a continuing panel survey conducted by Qualtrics on nationally-representative samples of adults (aged 18 or over) in France, Germany, Italy, Spain and Sweden. The Luxembourg panel is administered by TNS Ilres on a similar sample of individuals and using the same questionnaire. Stratification in Wave 1 ensured that each national sample was representative in terms of gender, region, and age. Other socioeconomic dimensions such as race were not considered in the process of stratification. Ethics approval was granted by the Ethics Review Panel of the University of Luxembourg. All research was carried out in accordance with relevant guidelines, and informed consent was obtained from all participants. Respondents complete an online questionnaire that takes approximately 25 min. The first six waves of the COME-HERE survey were conducted in late April, early June, early August, and late November 2020, and in early March and June 2021. Two more waves took place in October 2021 and February 2022, and two additional waves are planned in the remainder of 2022.

In the sixth survey wave, in June 2021, respondents were asked to indicate their vaccination status. There were three possible responses to the question “Have you been vaccinated against Covid-19?”: “Yes”, “No, but I plan to. I am waiting for my turn to come”, and “No, and I do not plan to”. We will refer to the participants who replied “No, and I do not plan to” as the vaccine-hesitant and the reference group will be made of those who replied “Yes” or “No, but I plan to. I am waiting for my turn to come”. The latter group are not vaccine-hesitant, as vaccination is not currently available to them (as in the WHO definition above).

The survey also collects detailed information on individuals’ living conditions and mental health during the pandemic, the recent changes and events in their lives, and standard sociodemographic characteristics such as age, gender, education, labour-force status, and country and region of residence^20^.

### Statistical analysis

To identify the most-important predictors of actual vaccine hesitancy, we estimate the following linear-probability model (estimation via either probit or logit models produces similar results):

1 $$VH_{ijt} = \beta_{1} D_{it} + \beta_{2} E_{it} + \beta_{3} H_{it} + \beta_{4} P_{it} + \beta_{5} Conf_{it - 1} + \beta_{6} COVID_{jt} + \lambda_{t} + \mu_{j} + \epsilon_{ijt} .$$

Here $VH_{ijt}$ is a dummy variable for individual *i* living in country *j* who was interviewed at time *t* who had not been vaccinated and did not plan to do so. The group to which the vaccine-hesitant are compared (for whom $VH_{ijt}$ is zero) are those who have already had the vaccine or who are planning to have it when their turn comes. $D_{it}$ is a vector of demographic characteristics (age, and dummy variables for being a woman, being partnered, having post-Secondary education, and having children living in the household). $E_{it}$ contains the following economic variables: equivalised (using the square root of household size) monthly net household income (in logs and converted across countries using Purchasing Power Parity information), labour-force status, and home-ownership (which latter acts as a proxy for wealth). We control for two health-related dummy variables in $H_{i}$: one for having at least one diagnosed physical health condition and the other for having tested positive for COVID-19 at least once since the beginning of the pandemic. The vector $P_{it}$ refers to the individual’s political opinions, measured via dummies for supporting a Right-wing or Left-wing party (with Centre parties being the reference group). $Conf_{it - 1}$ corresponds to the respondent’s degree of confidence in the government’s ability to handle the COVID-19 crisis (measured on a Likert scale from 1 to 7); to reduce concerns about common-method variance, the individual’s confidence in the government was taken from Wave 5 of COME-HERE that was fielded in March 2021. The correlation matrix between the variables in the vectors $D_{it}$, $E_{it}$, $H_{i}$, $P_{it}$ and $Conf_{it - 1}$ appears in Table A1 in the online Supplementary Information. All pairwise correlations are relatively weak.

Last, $COVID_{jt}$ includes aggregate variables at the country-day of interview level describing the evolution of the pandemic itself as well as the policy responses to COVID-19. Following the existing literature, we measure the former by the number of daily COVID-19 deaths per 100,000 inhabitants (as a 4-week average) and the latter by the Stringency Index (a 2-week average) from the Blavatnik School of Governance of the University of Oxford^21^. The Stringency Index is composed of the following nine sub-indices, measuring various aspects of containment policies: “school closing”, “workplace closing”, “cancellation of public events”, “restriction on gathering”, “public transport closing”, “stay-at-home requirements”, “restriction on internal movement”, “restriction on international travel” and “public information campaign”. This index is rescaled to range from 0 to 100: the higher the value of the Stringency Index, the more stringent is the country’s lockdown-style policy response to COVID-19. Last, $\lambda_{t}$ and $\mu_{j}$ are respectively day-of-interview and country fixed effects. All continuous variables are standardised to allow for the easy comparison of effect sizes across variables.

Equation (1) is estimated using information on respondents who have non-missing values for both the dependent and independent variables: this produces a sample of 4862 respondents. The total number of participants in COME-HERE Wave 6 (where vaccine hesitancy was measured) is 4899. We lose 37 observations due to missing values: five for vaccine hesitancy, and the remaining 32 from missing values for some of the independent variables.

Our observations are not weighted. Although the Wave 1 sampling protocol ensured national representativeness in terms of age, gender and region of residence, the remaining sample in Wave 6 may suffer from problems associated with selective attrition. We show in Table A2 of the online Supplementary Information that selective attrition does not seem to a major problem here, as our results are unchanged when we weight our observations using the standard Inverse Probability Weighting method.

The data from Wave 6 was collected during the course of June 2021, a period when the number of cases or deaths related to COVID-19 was at its lowest after the progression of the epidemic over the previous Winter. This downward trend appeared in all six countries in our sample. Correspondingly the values of the Stringency Indices also fell over this period, which reflects the relaxation of restrictions in the Summer of 2021. Despite the similarity in the profiles, it is important to note that there were significant differences in stringency levels between France, Germany, Italy and Spain on the one hand, and Sweden and Luxembourg on the other. The first group of countries had Stringency Indices of around 70 in June 2021, while in the second group the stringency scores were around 50.

### Ethics approval

This study was approved by the Ethics Review Panel of the University of Luxembourg.

## Results

### Descriptive statistics

The distribution of vaccination status in our estimation sample is as follows: 59% were already vaccinated in June 2021, a figure very close to that given in official statistics (see https://vaccinetracker.ecdc.europa.eu/public/extensions/COVID-19/vaccine-tracker.html#uptake-tab); 28% were not yet vaccinated, but planned to do so; and 13% were not vaccinated and did not plan to do so. This latter group are the vaccine-hesitant. Around half of the sample is female and the average age is 51. French, Italian and Spanish residents make up for 20–21% of the sample each, while Germans, Swedes and Luxembourgish represent around 13% of the observations. The descriptive statistics for the estimation sample appear in Table 1.

**Table 1:** Descriptive statistics of the estimation sample.

	Mean	SD	Min	Max
Vaccine hesitant	13.3%		0	100
Female	49.3%		0	100
Age	50.76	15.72	18	95
Living with a partner	63.0%		0	100
Post-secondary education	42.7%		0	100
Children in the household	27.8%		0	100
Equivalised Monthly net HH income (in logs)	7.46	0.64	5.22	9.36
Home-owner	69.0%		0	100
Employed	55.4%		0	100
Unemployed	4.9%		0	100
Out of labour force (working age)	10.7%		0	100
Retired	29.0%		0	100
Underlying health condition	32.7%		0	100
Ever tested positive for Covid-19	10.7%		0	100
Confidence in government	4.17	1.78	1	7
Political orientation: Left	24.0%		0	100
Political Orientation: Centre	49.7%		0	100
Political orientation: Right	26.3%		0	100
Number of daily deaths/100,000 inhabitants (4-week average)	0.17	0.07	0.05	0.28
Stringency index (2-week average)	61.8	9.26	43.3	74.3
France	19.7%		0	100
Germany	14.4%		0	100
Italy	20.0%		0	100
Spain	21.2%		0	100
Sweden	11.7%		0	100
Luxembourg	12.9%		0	100

These figures refer to our estimation sample. Percentages are reported instead of decimal means for dummy variables. “Vaccine hesitant” are those who replied “No, I do not plan to” to the question “Have you been vaccinated against Covid-19?”. “Post-Secondary Education” is a dummy for individuals with a college degree. “Confidence in Government” is the respondent’s degree of confidence in the government’s ability to handle the COVID-19 crisis (measured on a Likert scale from 1 to 7). “Stringency Index” is an index produced by the Blavatnik School of Governance of the University of Oxford composed of nine sub-indices measuring the various aspects of containment policies, and has been rescaled to range from 0 to 100.

### Vaccine hesitancy by groups

**Figure 1:**
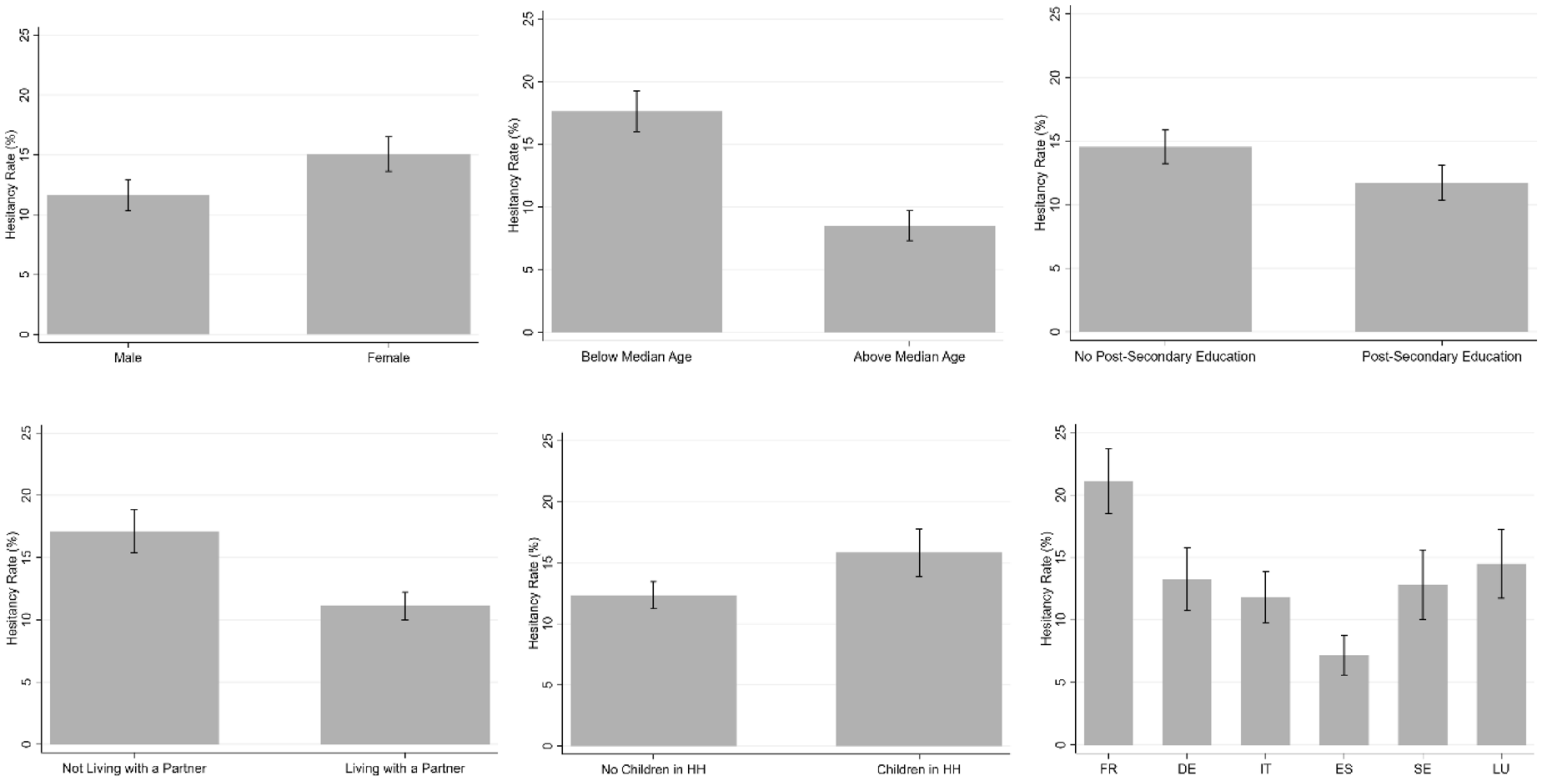
Vaccine hesitancy: demographic groups and countries. *Notes*: These figures refer to our estimation sample. The grey bars show the percentage of vaccine-hesitant in each group, and the intervals refer to the 95% confidence intervals.

**Figure 2:**
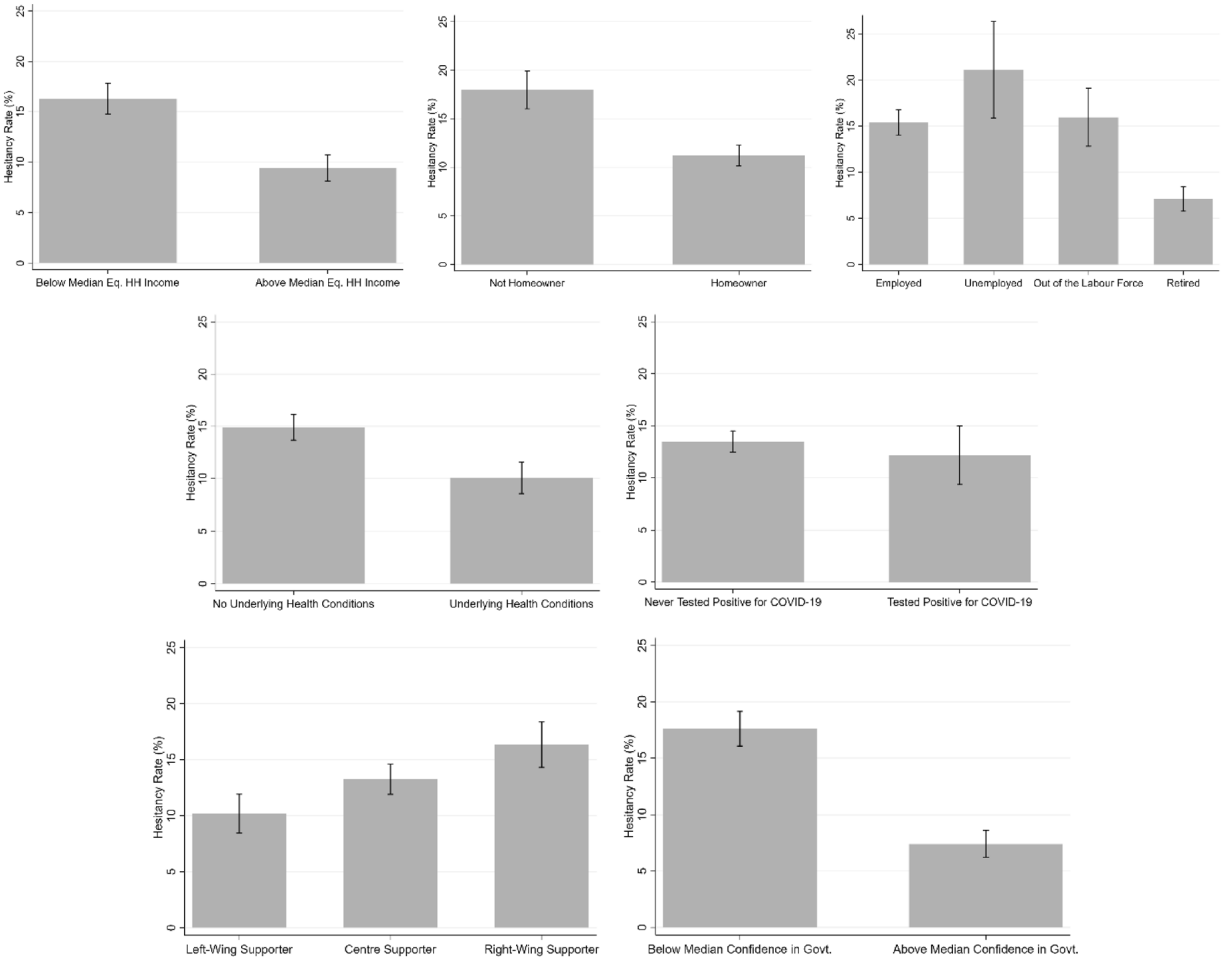
Vaccine hesitancy: other individual characteristics. *Notes*: These figures refer to our estimation sample. The grey bars show the percentage of vaccine-hesitant in each group, and the intervals refer to the 95% confidence intervals.

Figures 1 and 2 depict the average hesitancy rates for different groups of respondents. In Fig. 1, hesitancy is significantly higher for women, the younger and the less-educated; it is also higher for individuals without a partner and those with children in the household. The six countries seem to split into three groups: France, where hesitancy is the highest (at 21%); Germany, Italy, Sweden and Luxembourg, where the hesitancy rates lie between 13 and 15%; and last Spain with the lowest figure of 7%. In Fig. 2, there are also significant differences in hesitancy by economic variables: the economically-vulnerable (below median equivalised monthly net household income, not homeowners, and the unemployed) have significantly higher hesitancy rates. The retired are one of the groups with the lowest hesitancy (7%). Health vulnerability works in the opposite direction to economic vulnerability, with the hesitancy rate being lower for those with underlying health conditions. There is no difference in hesitancy by having tested positive for COVID-19. There is a distinct political slope in vaccine hesitancy, ranging from 10% of those with Left-Wing views to over 15% of those with Right-wing views. One of the largest contrasts is with respect to confidence in the government’s ability to handle the COVID-19 crisis: those with low confidence are twice as likely to be vaccine-hesitant than those with greater confidence.

### Predictors of vaccine hesitancy

We now turn to regression analysis to disentangle the separate effects of the different factors in Figs. 1 and 2: the results appear in Table 2. We introduce groups of independent variables sequentially in order to see whether the right-hand variables in the estimation model have a direct or rather a mediated effect on vaccine hesitancy.

**Table 2:** The predictors of vaccine hesitancy—ordinary least squares results.

	Vaccine-hesitant				
	(1)	(2)	(3)	(4)	(5)
Female	0.011	0.006	0.004	0.011	0.011
	(0.010)	(0.010)	(0.010)	(0.010)	(0.012)
Age^S^	− 0.052***	− 0.040***	− 0.038***	− 0.035***	− 0.035***
	(0.005)	(0.007)	(0.007)	(0.007)	(0.008)
Post-secondary education	− 0.047***	− 0.034***	− 0.034***	− 0.037***	− 0.037***
	(0.010)	(0.010)	(0.010)	(0.010)	(0.008)
Living with a partner	− 0.042***	− 0.028**	− 0.028**	− 0.026**	− 0.026**
	(0.011)	(0.011)	(0.011)	(0.011)	(0.009)
Children in the household	0.014	0.006	0.007	0.009	0.009
	(0.012)	(0.012)	(0.012)	(0.012)	(0.013)
Equivalised monthly net HH income (in logs)^S^		− 0.031***	− 0.032***	− 0.029***	− 0.029***
		(0.005)	(0.006)	(0.005)	(0.006)
Home-owner		− 0.033***	− 0.033***	− 0.033***	− 0.033***
		(0.011)	(0.011)	(0.011)	(0.011)
**Employment status (Ref. = Employed)**					
Unemployed		0.028	0.028	0.027	0.026
		(0.023)	(0.023)	(0.023)	(0.022)
Out of labour force (working age)		− 0.030*	− 0.029*	− 0.020	− 0.020
		(0.017)	(0.017)	(0.016)	(0.015)
Retired		− 0.027*	− 0.026*	− 0.018	− 0.018
		(0.015)	(0.015)	(0.015)	(0.017)
Underlying health condition			− 0.028***	− 0.031***	− 0.031***
			(0.010)	(0.010)	(0.008)
Ever tested positive for Covid-19			− 0.014	− 0.016	− 0.016
			(0.015)	(0.015)	(0.014)
Confidence in government^s^				− 0.060***	− 0.060***
				(0.005)	(0.007)
**Political orientation (Ref. = Centre)**					
Political orientation: Left				0.005	0.004
				(0.012)	(0.012)
Political orientation: Right				0.029**	0.029*
				(0.012)	(0.018)
Number of daily deaths/100,000 inhabitants					− 0.008
(4-week average)^S^					(0.039)
Stringency index (2-week average)^S^					− 0.050
					(0.056)
**Country dummies (Ref. = France)**					
Germany	− 0.083***	− 0.085***	− 0.085***	− 0.065***	0.032
	(0.017)	(0.017)	(0.017)	(0.017)	(0.105)
Italy	− 0.116***	− 0.122***	− 0.122***	− 0.095***	0.001
	(0.015)	(0.016)	(0.016)	(0.015)	(0.121)
Spain	− 0.142***	− 0.146***	− 0.142***	− 0.138***	− 0.107***
	(0.015)	(0.015)	(0.015)	(0.015)	(0.035)
Sweden	− 0.096***	− 0.107***	− 0.107***	− 0.098***	− 0.071**
	(0.018)	(0.018)	(0.018)	(0.018)	(0.033)
Luxembourg	− 0.047	− 0.023	− 0.017	0.013	− 0.029
	(0.033)	(0.033)	(0.033)	(0.032)	(0.087)
Observations	4862	4862	4862	4862	4862
Adjusted R^2^	0.052	0.062	0.063	0.095	0.095

All regressions include day-of-interview fixed effects. All continuous independent variables are standardised: these are indicated by a ^S^ next to the variable name. Standard errors in column (5) are clustered at the country*day of the interview level.

*, **, and *** indicate respectively significance at the 10%, 5% and 1% levels.

In all of the columns starting from (1), where we only control for demographic characteristics, to the full model in column (5), the estimated coefficients on age, being partnered, and post-Secondary education are all negative, significant at the 5% level at least, and of similar size. A rise in age of one standard deviation (just under 16 years from Table 1) is associated with higher vaccine hesitancy of around 4% points: this figure is to be compared to the mean incidence of vaccine-hesitancy of 13 percent in our sample. The use of a quadratic age specification or dummies for age categories to account for non-linear effects produces similar effect sizes. Individuals with post-Secondary education are estimated to have 3.7% points lower vaccine hesitancy, as compared to the reference group of at most Secondary education. The partnered are less vaccine-hesitant, but there is no relationship with having children in the household. The insignificance of the latter may reflect its correlation with age: if we do not control for age, we do find that individuals with children in the household are significantly less vaccine-hesitant. The fall in vaccine hesitancy with age in all specifications may be explained by a number of phenomena. This may first reflect the well-known finding that risk aversion is lower for the older^22–26^, second that the elderly had priority access to vaccines, and third that the number of Doctor visits generally rises with age (and it may be that Doctors recommended taking the vaccine). Vaccine hesitancy is equally lower for the educated and those with larger families, again consistent with the existing correlations with risk aversion^27^. This education result is not COVID-19 specific, as previous work has underlined that the educated are in general less vaccine-hesitant^28^. It is notable that there is no gender difference in vaccine hesitancy in Table 2, as opposed to the significant difference in the raw data in Fig. 1. This suggests that the raw gender gap in Fig. 1 mostly reflects the influence of confounders or mediators.

In columns (2–5), both income and home-ownership are consistently positively associated with vaccine acceptance. The size of the correlation with home-ownership is similar to that for post-Secondary education, while the estimated coefficient of − 0.03 on log income implies that a one-standard deviation increase in log income (that is, an increase of 64%) would reduce vaccine hesitancy by 2% points. Attitudes towards vaccines during the pandemic are then consistent with those found in past work, where low income was found to be associated with greater concerns about the safety and necessity of vaccines^29–31^. This pattern does not reflect age or education, as these are controlled for in the multivariate analysis. It may rather reflect that the cost of illness is higher for the well-off (in terms of lost income), or that some leisure activities that are associated with income, such as international travel, require proof of vaccination. In Fig. 2, the unemployed and the retired had the highest and the lowest hesitancy rates respectively. In Table 2, almost all of the gaps between different labour-force statuses are explained by the associated differences in income, education and other demographic variables.

In columns (3–5), we see that respondents with diagnosed physical health conditions (e.g. cancer, diabetes, or heart disease) are also less likely to be vaccine-hesitant. This may reflect the health consequences of COVID-19 infection, rather than any confounding effect of, say, income or education. In alternative specifications (see Table A3 in the online Supplementary Information), we included measures of mental health, such as anxiety and depression, but none of these attracted significant estimates when included. Vaccine hesitancy is then more correlated with physical than mental health. As in the raw data, we find no significant effect of having tested positive for COVID-19.

Columns (4) and (5) add the respondent’s degree of confidence in the Government, and the political-support dummies. Confidence in the government explains most of the improvement in the quality of the fit (as revealed by the higher Adjusted R^2^ figure at the foot of the table): a one standard-deviation rise in confidence in the government is associated with a reduction in vaccine hesitancy of 6% points. Last, those with Right-wing attitudes are more likely to be vaccine-hesitant. The differences in vaccine hesitancy by political position and confidence in the regressions are consistent with those in Fig. 2. Confidence in the health services has been shown to reduce hypothetical vaccine hesitancy^13^; we here find that it plays the same role in actual vaccine hesitancy. Those who identify as Right-wing are more vaccine-hesitant in our data, consistent with conservatism being associated with science scepticism (see Rujtens and colleagues^32^ for a detailed literature review). It can also be argued that the pandemic policies to tackle the spread of infection have reduced individual liberties, counter to Conservative values of individual freedom and limited government intervention^33^. Note that the standard errors remain almost unchanged with the successive introduction of the new independent variables across the different columns of Table 2: this is consistent with the weak correlations in Table A1 in the online Supplementary Information, and helps to reassure us that there are no major problems with multicollinearity.

Column (5) of Table 2 introduces the pandemic variables: the 4-week average number of daily deaths/100,000 inhabitants and the 2-week average Stringency Index. Conditional on all of the other explanatory variables, these have no significant impact on vaccine attitudes. This may reflect low statistical power, as they only vary at the country*day of interview level, and we control for country dummies (so that identification only comes from within-country variation in the pandemic at the different interview days of the June COME-HERE survey wave). Note that the standard errors are clustered at the country*day of the interview in column (5).

Last, the estimated coefficients on the country dummy variables show the residual variation in country vaccine attitudes. The reference country is France (the country with the highest hesitancy rate in Fig. 2). Following the introduction of the country-specific COVID-19 variables in the last column, many of the substantial differences between countries in Fig. 1 disappear: there are now no differences in vaccine hesitancy between France, Germany, Italy and Luxembourg. However, the hesitancy rates in Spain and Sweden remain significantly different from zero even after controlling for demographic, attitudinal and pandemic variables. Long-standing cultural and institutional country differences may partially explain these patterns in the country coefficients: according to data from the 2018 Wellcome Global Monitor (a part of the Gallup World Poll), pre-pandemic Spain and Sweden already figured amongst the European countries with higher population percentages who believed that vaccines were safe.

As the continuous independent variables are standardised, the estimated coefficients reveal the strongest predictors of vaccine hesitancy. This comparison reveals that confidence in the government’s ability to handle the COVID-19 crisis is the most important factor lying behind vaccine hesitancy: a one standard-deviation fall in confidence in the government leads to greater vaccine hesitancy that is 50% above baseline hesitancy. The effects of age, education, income, wealth, health and political attitudes are all around half of this level.

## Discussion

In European surveys between September 2020 and January 2021, the hypothetical vaccine-hesitancy rate lay between 18 and 65%^8,10,13^. However, the average actual vaccine hesitancy rate in our estimation sample is far below these figures, at 13%. There are a number of potential explanations of this gap. Individuals may be more risk-averse in real life than in hypothetical situations^34,35^. Experimental evidence also suggests that stated and revealed preferences may differ when choices involve social concerns^36^, and lower actual vaccine hesitancy may reflect the saliency of these social concerns in real-life decisions.

The pattern of average hesitancy rates in our raw data (higher for women, the younger and the less-educated, for example) is in line with that found in the existing literature based on hypothetical choices^8–15,29–31,33^. Our multivariate analysis reveals that most of the raw gaps continue to hold when keeping constant the influence of confounders and mediators. In all specifications, vaccine hesitancy consistently falls with age, education, partnership, income, wealth (the latter being proxied by home-ownership), pre-existing health conditions, confidence in the government and political orientations not leaning to the Right.

However, not all of the differences in means are found in the multivariate analysis. In particular, there is no sex difference in vaccine hesitancy in the regression analysis, whereas there was in Fig. 1. It turns out that the raw gender difference is entirely explained by age (men in the estimation sample are over 5 years older on average than women). Age also confounds the raw gaps by retirement and the presence of children. The greater raw vaccine hesitancy of the unemployed we found is on the contrary not confounded by age (there is no statistically-significant age difference between the employed and unemployed), but is rather mediated by income. Were the employed and unemployed to be in households with the same level of equivalised monthly net household income they would report the same level of vaccine hesitancy. Last, only the hesitancy rates in Spain and Sweden remain statistically different from zero even after controlling for demographic, attitudinal and pandemic variables.

Beyond the fact that our analysis allows us to confirm whether a given variable has significant predictive power for vaccine hesitancy, the estimated coefficients also reveal the strongest predictors of vaccine hesitancy. Confidence in the government’s ability to handle the COVID-19 crisis is the most important factor behind vaccine hesitancy. The effects of age, education, income, wealth, health and political attitudes are all around half of the size.

These gaps between groups in terms of actual vaccine hesitancy are always lower than those found in hypothetical vaccine questions. For instance, the raw difference in vaccine hesitancy between men and women in hypothetical choices ranges from 6 to 12% points, but our gender gap in actual vaccine hesitancy is 3.4% points^8–15^. The same conclusion applies to age and education, where the mean stated vaccine hesitancy differences between the youngest and oldest age groups and between the lowest and highest education groups are twice as large as those we find in revealed vaccine hesitancy^8,14^. The finding of smaller gaps between sociodemographic groups (such as age, education, gender and income) extends to the results from the regression analyses^8–15^. In multivariate regressions, women are more vaccine-hesitant in hypothetical data, whereas we find no significant difference in our analysis of actual preferences. Smaller actual vaccine-hesitancy gaps are also found for variables such as income, education and confidence^8–15^. Consequently, while they are often similar in sign, the size of the correlations with observed variables may differ considerably between actual and hypothetical choices. This is consistent with past evidence on the predictors of stated trust and the trust that is revealed in actual choices in laboratory experiments^37^.

These results have important implications for vaccination policies. We find no relationship between policy stringency and vaccine hesitancy, so that relaxing restrictions on movement has not affected vaccine take-up. Our results also identify the groups that are *ex post* vaccine-hesitant, and which policy-makers may wish to target in order to increase vaccination rates and the chance of reaching herd immunity. They also underline that hypothetical attitudinal questions often seem to be able to pick out the groups who will indeed turn out to be more vaccine-hesitant in practice, even though they may not be a good guide to the size of the gaps between groups.

## Supplementary Information

Supplementary Information.

## Data Availability

The data is not publicly available. However, the code used in the analysis will be made available.
