# Supplementary material for: Characteristics associated with COVID-19 vaccine hesitancy
Source: Sci Rep. 2022 Jul 20;12:12435. doi: 10.1038/s41598-022-16572-x (PMC9298705; doi:10.1038/s41598-022-16572-x)
Supplement: Supplementary file 1 — Supplementary Information. [file 41598_2022_16572_MOESM1_ESM.pdf]

Supplementary Information for:

Characteristics Associated with Vaccine Hesitancy

Liyousew G. Borga[1], Andrew E. Clark[1,2], Conchita D'Ambrosio[1], Anthony Lepinteur[1*]

[1] University of Luxembourg, Department of Behavioural and Cognitive Sciences, L-4366 Esch-sur-Alzette

[2] Paris School of Economics – CNRS, 75014 Paris

[*] Corresponding author. Email: anthony.lepinteur@uni.lu

Table A1: Matrix of Correlations – Individual Control Variables

	Female	Age	Post-Sec. Educ.	Living with a Partner	Children in the HH	HH Income	Home - owner	Employed	Unempl.	OLF	Retired	Underl. Health Condition	Covid-19 Positive	Conf. in Govt.	Pol. Or.: Center	Pol. Or.: Right	Pol. Or.: Left
Female	1																
Age	-0.17	1															
Post-Secondary Education	-0.04	-0.13	1														
Living with a Partner	-0.11	0.20	0.02	1													
Children in the Household	0.05	-0.33	0.11	0.21	1												
HH Income	-0.16	0.18	0.20	0.20	-0.08	1											
Home-owner	-0.06	0.09	0.09	0.18	0.11	0.17	1										
Employed	-0.01	0.46	0.22	0.02	0.34	0.10	0.03	1									
Unemployed	0.06	-0.09	-0.04	-0.10	-0.03	-0.19	-0.07	-0.25	1								
OLF	0.18	-0.21	-0.12	-0.09	-0.02	-0.18	-0.06	-0.39	-0.08	1							
Retired	-0.14	0.69	-0.35	0.08	-0.35	0.11	0.04	-0.71	-0.14	-0.22	1						
Underlying Health Condition	-0.07	0.12	-0.00	0.05	-0.01	-0.01	0.03	-0.08	-0.01	-0.02	0.10	1					
Covid-19 Positive	-0.02	-0.04	0.02	0.01	0.07	002	0.02	0.05	-0.03	0.01	-0.05	0.05	1				
Confidence in Government	0.03	0.06	-0.05	0.03	-0.01	0.06	0.03	-0.07	-0.04	0.04	0.07	-0.00	-0.00	1			
Political Orientation: Center	0.05	-0.03	-0.05	-0.01	0.00	-0.02	-0.03	0.00	-0.01	0.04	-0.03	-0.01	-0.01	0.00	1		
Political Orientation: Left	0.00	0.04	0.02	-0.02	-0.05	-0.00	-0.00	-0.04	0.04	-0.00	0.03	-0.00	-0.02	0.13	-0.56	1	
Political Orientation: Right	-0.06	0.00	0.03	0.04	0.04	0.02	0.04	0.04	-0.03	-0.05	0.00	0.01	0.03	-0.13	-0.59	-0.34	1

Notes: These figures refer to our estimation sample. These are pairwise correlations.

Table A2: The Predictors of Vaccine Hesitancy – Ordinary Least Squares Results with IPW

	Vaccine-Hesitant				
	(1)	(2)	(3)	(4)	(5)
Female	0.010	0.005	0.004	0.011	0.011
	(0.010)	(0.010)	(0.010)	(0.010)	(0.013)
Age^S	-0.050***	-0.039***	-0.037***	-0.034***	-0.034***
	(0.005)	(0.007)	(0.007)	(0.007)	(0.009)
Post-Secondary Education	-0.045***	-0.034***	-0.033***	-0.036***	-0.036***
	(0.010)	(0.010)	(0.010)	(0.010)	(0.009)
Living with a Partner	-0.042***	-0.029**	-0.029**	-0.027**	-0.027***
	(0.011)	(0.011)	(0.011)	(0.011)	(0.010)
Children in the Household	0.020*	0.011	0.011	0.015	0.014
	(0.012)	(0.012)	(0.012)	(0.012)	(0.014)
Equivalised Monthly Net HH Income (in logs)^S		-0.034***	-0.035***	-0.031***	-0.031***
		(0.006)	(0.006)	(0.006)	(0.006)
Home-owner		-0.036***	-0.036***	-0.035***	-0.035***
		(0.012)	(0.012)	(0.011)	(0.012)
Unemployed		0.019	0.020	0.019	0.018
		(0.023)	(0.023)	(0.023)	(0.025)
Out of Labour Force (Working Age)		-0.043***	-0.043***	-0.033**	-0.032**
		(0.016)	(0.016)	(0.016)	(0.016)
Retired		-0.032**	-0.031*	-0.022	-0.021
		(0.016)	(0.016)	(0.016)	(0.018)
Underlying Health Condition			-0.028***	-0.031***	-0.031***
			(0.011)	(0.011)	(0.009)
Ever Tested Positive for Covid-19			-0.014	-0.017	-0.017
			(0.016)	(0.015)	(0.016)
Confidence in Government^S				-0.063***	-0.063***
				(0.005)	(0.007)
Political Orientation: Left				-0.002	-0.003
				(0.012)	(0.014)
Political Orientation: Right				0.028**	0.028
				(0.012)	(0.018)
Number of daily deaths/100,000 inhabitants					0.011
(4-week average)^S					(0.042)
Stringency Index (2-week average)^S					-0.037
					(0.061)
Germany	-0.092***	-0.092***	-0.092***	-0.072***	0.002
	(0.017)	(0.017)	(0.017)	(0.017)	(0.113)
Italy	-0.140***	-0.144***	-0.144***	-0.116***	-0.056
	(0.017)	(0.017)	(0.017)	(0.017)	(0.127)
Spain	-0.158***	-0.161***	-0.158***	-0.153***	-0.106***
	(0.016)	(0.016)	(0.016)	(0.016)	(0.040)
Sweden	-0.110***	-0.119***	-0.118***	-0.110***	-0.070*
	(0.018)	(0.018)	(0.018)	(0.018)	(0.037)
Luxembourg	-0.056*	-0.029	-0.023	0.009	0.003
	(0.032)	(0.032)	(0.032)	(0.032)	(0.092)
Observations	4862	4862	4862	4862	4862
Adjusted R^2	0.053	0.064	0.065	0.097	0.097

Notes: All regressions include day-of-interview fixed effects. All continuous independent variables are standardised: these are indicated by a ^{S} next to the variable name. Standard errors in column (5) are clustered at the country*day of the interview level. *, **, and *** indicate respectively significance at the 10%, 5% and 1% levels. Observations are weighted using a standard IPW.

Table A3: The Predictors of Vaccine Hesitancy – Ordinary Least Squares Results with Mental-Health Variables

	Vaccine-Hesitant				
	(1)	(2)	(3)	(4)	(5)
Female	0.011	0.006	0.004	0.012	0.012
	(0.010)	(0.010)	(0.010)	(0.010)	(0.013)
Age^{S}	-0.052***	-0.040***	-0.038***	-0.036***	-0.036***
	(0.005)	(0.007)	(0.007)	(0.007)	(0.008)
Post-Secondary Education	-0.047***	-0.034***	-0.034***	-0.037***	-0.037***
	(0.010)	(0.010)	(0.010)	(0.010)	(0.008)
Living with a Partner	-0.042***	-0.028**	-0.027**	-0.026**	-0.026***
	(0.011)	(0.011)	(0.011)	(0.011)	(0.009)
Children in the Household	0.014	0.006	0.007	0.010	0.009
	(0.012)	(0.012)	(0.012)	(0.012)	(0.013)
Equivalised Monthly Net HH Income (in logs)^{S}		-0.031***	-0.031***	-0.029***	-0.029***
		(0.005)	(0.006)	(0.005)	(0.006)
Home-owner		-0.033***	-0.033***	-0.033***	-0.033***
		(0.011)	(0.011)	(0.011)	(0.011)
Unemployed		0.028	0.028	0.027	0.026
		(0.023)	(0.023)	(0.023)	(0.022)
Out of Labour Force (Working Age)		-0.030*	-0.029*	-0.019	-0.019
		(0.017)	(0.017)	(0.016)	(0.015)
Retired		-0.027*	-0.027*	-0.018	-0.018
		(0.015)	(0.015)	(0.015)	(0.017)
Underlying Health Condition			-0.029***	-0.030***	-0.030***
			(0.011)	(0.010)	(0.008)
Ever Tested Positive for Covid-19			-0.014	-0.015	-0.015
			(0.015)	(0.015)	(0.014)
GAD-7 (Anxiety)^{S}			-0.006	-0.011	-0.012
			(0.008)	(0.008)	(0.008)
PHQ (Depression)^{S}			0.008	0.007	0.008
			(0.008)	(0.008)	(0.007)
Confidence in Government^{S}				-0.061***	-0.061***
				(0.005)	(0.007)
Political Orientation: Left				0.005	0.004
				(0.012)	(0.012)
Political Orientation: Right				0.030**	0.030*
				(0.012)	(0.018)
Number of daily deaths/100,000 inhabitants					-0.008
(4-week average)^{S}					(0.039)
Stringency Index (2-week average)^{S}					-0.051
					(0.056)
Germany	-0.083***	-0.085***	-0.085***	-0.065***	0.035
	(0.017)	(0.017)	(0.017)	(0.017)	(0.105)
Italy	-0.116***	-0.122***	-0.122***	-0.093***	0.005
	(0.015)	(0.016)	(0.016)	(0.016)	(0.121)
Spain	-0.142***	-0.146***	-0.142***	-0.137***	-0.106***
	(0.015)	(0.015)	(0.015)	(0.015)	(0.035)
Sweden	-0.096***	-0.107***	-0.107***	-0.099***	-0.072**
	(0.018)	(0.018)	(0.018)	(0.018)	(0.034)
Luxembourg	-0.047	-0.023	-0.016	0.014	-0.029
	(0.033)	(0.033)	(0.033)	(0.032)	(0.087)
Observations	4862	4862	4862	4862	4862
Adjusted R^2	0.052	0.062	0.063	0.095	0.095

Notes: All regressions include day-of-interview fixed effects. All continuous independent variables are standardised: these are indicated by a ^{S} next to the variable name. Standard errors in column (5) are clustered at the country*day of the interview level. *, **, and *** indicate respectively significance at the 10%, 5% and 1% levels.
